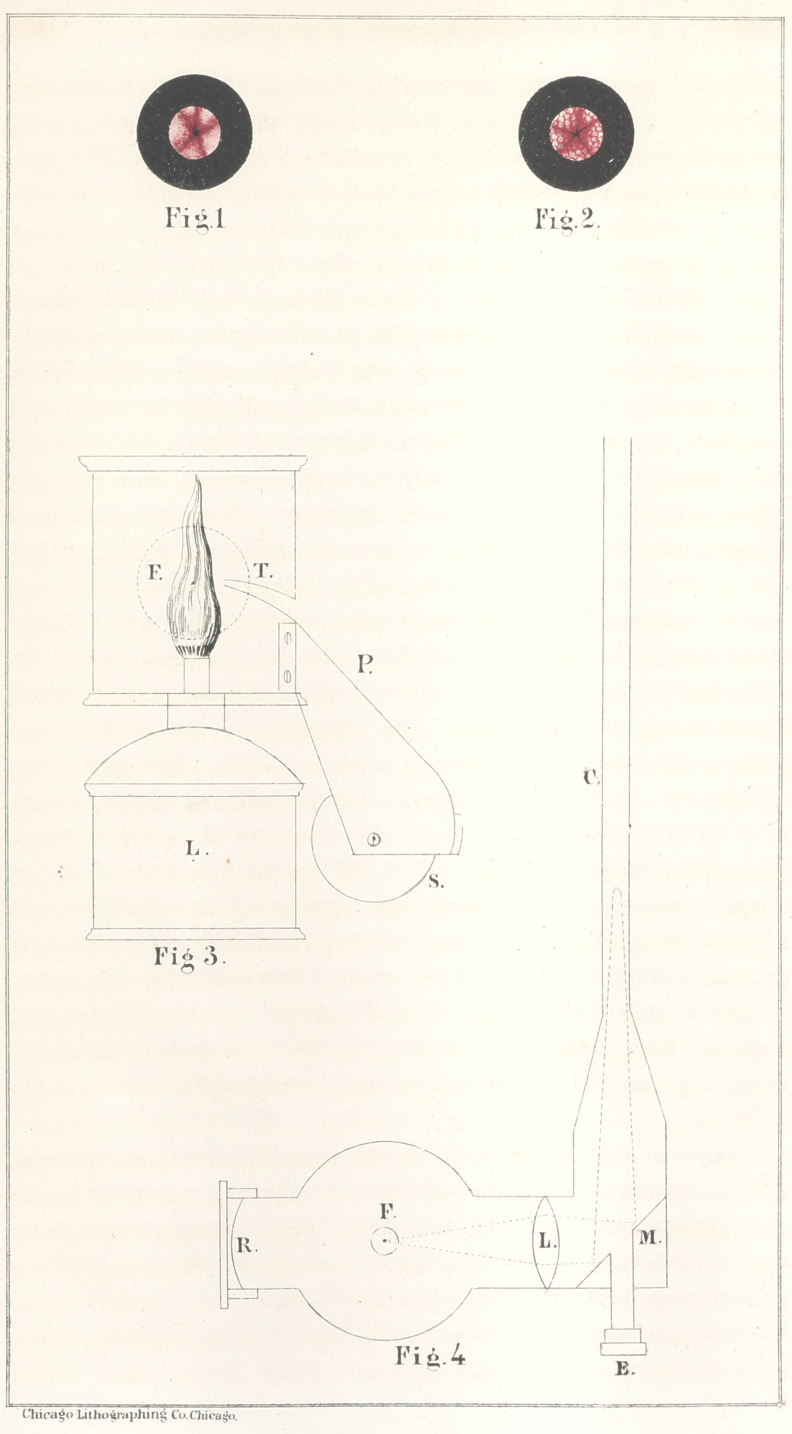# Improved Form of the Endoscope

**Published:** 1868-08

**Authors:** E. Andrews

**Affiliations:** Prof. of Principles and Practice of Surgery, Chicago Medical College


ARTICLE XXVIII.

### IMPROVED FORM OF THE ENDOSCOPE.

By E. ANDREWS, M.D., Prof. of Principles and Practice of Surgery, Chicago Medical College.

[Read to the Illinois State Medical Society, May 20, 1868.]

The object of the endoscope is the examination of cavities of the body not accessible to ordinary vision. The form best known in this country is the one invented by Desormeaux, of Paris. It consists essentially of three parts, *viz.:* the lantern, the perforated mirror, and the tube. The lantern is an enclosed lamp, filled with burning fluid, having on one side a concave reflector (R Fig. 4), whose function is to send back the rays which fall upon it, so as to increase the light thrown in the opposite direction. The light, thus re-enforced, falls upon a lens (L, Fig. 4), which condenses it upon the perforated plane mirror (M, Fig. 4). The latter being set at an angle of forty-five degrees, throws the light in a condensed beam into the tube (C, Fig. 4), and strongly illuminates the interior of any cavity into which the tube is inserted, rendering it visible to the eye placed at E, Fig. 4, where it looks through the perforation of the mirror directly into the tube.

The principle is plain, but the practical difficulty is, that unless the apparatus be in perfect order, the light fails to reach the bottom of such a deep, narrow tube in sufficient strength to illuminate and render visible the membranes at its extremity. So great is this difficulty that I apprehend that the iustrument, in the form devised by the European inventors, will never come into extensive practical use. In order to overcome this evil, and produce an instrument which will display its objects when under such management as it would be likely to get in ordinary hands, it is necessary to have a more intense light than can be obtained by any common lamp. I have, therefore, devised an apparatus for burning a magnesium wire in the flame of the lamp, which gives an illumination of such intensity that it can only be compared to the blaze of the noonday sun. This attachment is very simple, and is represented in the sectional view, Fig. 3, in which L is the lamp, and F the flame. S is a revolving metallic spool, on which the wire is wound; and P represents an inclined plane, along which the wire passes. Through an opening in the side of the lantern the wire enters the tube T, which conducts it to the flame of the lamp. If, now, the thumb be pressed upon the inclined plane, and then moved upward, it will slide the wire forward, and cause it to enter the flame, where it burns with a brilliancy far superior to that of the best lamp. I at first intended to have the wire advanced to the flame by means of springs and wheels, but a little experimentation convinced me that this would complicate the apparatus too much, and that it was better to simply slide forward the wire by the pressure of the thumb. The spool itself may be dispensd with, without inconvenience, the observer simply taking a piece of the wire and inserting it without winding it upon anything.

The endoscope is adapted to the inspection of all deep cavities and mucous passages into which a straight tube may be passed. It may be used for examining the ear, the rectum, and the bladder, as well as the interior of abscesses. It may be passed into the track of bullet wounds, to distinguish broken bones from impacted balls, and to detect pieces of cloth and other foreign bodies. It has been even inserted through punctures into ovarian tumors, to examine their interior. Calculi in the bladder have also been detected by it. Its principal practical use, however, is the examination of the interior of the urethra, as a means of diagnosing its different diseases, and of ascertaining the progress of the cure.

In order to understand its usefulness for this purpose, a few remarks on the pathology of the urethra are necessary. The healthy urethra is lined with a smooth membrane, of a light pink hue, like that of most other mucous canals. When collapsed, its walls fall together in five or six longitudinal folds, so that, seen through the endoscope, the folds, prolapsed over the end of the tube, present a radiated form, as shown in Figs. 1 and 2. If the membrane be inflamed or congested, it presents a deeper red; and if anæmic, it is seen to be abnormally pale. The most important revelation of the endoscope is the complete establishment of the fact that the mucous membrane of the urethra, like the conjunctiva oculi, is subject to a granular inflammation, and that this disease constitutes the true lesion in many chronic gonorrhœas. Some years ago this idea was broached in Europe, and experiments were made on abandoned women, hired for that purpose, which showed that granular ophthalmia might be inoculated into the urethra, and there produce a perfectly similar disease, which could not be distinguished from chronic gonorrhœa. The want of a proper instrument for inspection, however, rendered the demonstration rather difficult and unsatisfactory, and this important discovery fell into obscurity. Desormeaux's instrument has brought this fact again into notice, and given good reasons to believe in the following propositions:

1. True chronic gonorrhœa, is a granular inflammation, identical in nature with the granular inflammations of the eyelids, of the cervix uteri and of the larynx; and one may be produced from the other by inoculation.

2. When this disease exists, it has no tendency to spontaneous recovery, but will last indefinite years unless treated, and will communicate contagion as long as it continues.

This disease often persists long after the patient believes himself or herself free from the clap, and hence the frequency with which men contract gonorrhœa from females whom they believe, and who believe themselves, to be free from disease.

The chief use of the endoscope is the diagnosis and treatment of this disease. In order to employ it to advantage, a variety of tubes are required, but the smallest should at least equal a No. 10 catheter. The tube is first detached from the lamp,

Fig.1

Fig.2.

Fig 3.

Fig.4

Chicago Lithographing Co. Chicago.

oiled, and a core is placed in it, which projects in a rounded extremity beyond the tip, to facilitate the entrance. If the urethra is too contracted, it must first be dilated. The tube should be passed pretty nearly to the prostate gland, when the core is withdrawn and a pledget of cotton pushed through it into the urethra beyond, to prevent the flow of mucus or other fluids into the tube. This pledget may be left there, to be expelled by the next passage of urine. Attaching the lamp, and applying the eye to the instrument, the tube should be gradually withdrawn, so that every part of the canal successively prolapses over the extremity of the tube. At the same time the magnesium wire is kept burning, and thus the whole interior of the urethra is inspected. The appearance of the granular disease thus seen is represented in Fig. 2; while the healthy urethra is represented by Fig. 1.

The applications to be used here are precisely those found most useful in granular conjunctivitis, *viz.*, nitrate of silver, sulphate of copper, tannin, alum, acetate of lead, subnitrate of bismuth, carbolic acid, etc. An injection of one part of carbolic acid, dissolved in three parts of linseed oil, frequently has a powerful effect in arresting the suppurative discharge, though it is rather severe treatment. In Paris, at the present time, great advantage is claimed for the use of powders blown in through the tube and dusting the interior of the urethra. In my own practice, I commonly carry in the powders on a pledget of cotton wound on the end of a wire. For this purpose, nitrate of silver, sulphate of copper, and various other medicaments, may be finely pulverized, along with subnitrate of bismuth, using a greater or lesser proportion, according to the strength required.

Granular inflammation of the urethra, like the same disease on the eyelids and in the cervix uteri, is extremely slow and obstinate—several months, and often a year or more, being consumed in the cure. As long as the endoscope shows any of the granulations remaining, so long the disease exists, and its contagiousness continues. During all this time the discharge may be scarcely worthy of notice, and not at all purulent, but a slight irritation serves to rouse the granulations into activity, so that the patient, without being subjected to any new contagion, may have all the symptoms of a new attack of acute gonorrhœa. Chronic urethretis and metritis, like granular conjunctivitis, may be communicated in a quiescent form, the granulations springing up gradually, accompanied by a slight irritation, without the patient ever passing through the symptoms of ordinary acute clap. Hence, many persons will give gonorrhœa to others who are utterly unaware that they have ever had the disease themselves. This is one reason why the weekly medical examinations of strumpets, under the license systems of France and Prussia, have proved so worthless for restraining the spread of venereal disease. The quiescent form of the disease escapes the notice of the examiner, who never looks for granulations, but only for the more noticeable phenomena of ordinary gonorrhœa and syphilis. Hence, a large portion of the prostitutes, who weekly receive their certificate of health, are really diseased, and become chronic centres of contagion, as many a verdant Englishman and American has found out to his cost, who, trusting to the fancied security of the governmental inspections, has indulged in Parisian licentiousness.

I stated above that granulur urethretis has scarcely any tendency to spontaneous cure. I have seen cases of 20 years' standing which were still in full activity. There is, however, at length, a sort of natural termination, which is reached by some patients in a few years, but by others not in a lifetime. The tendency of the granular disease is to gradually infiltrate the mucous membrane with permanently organized plastic lymph. This, like all other new tissue, undergoes contraction, which gradually compresses the contained bloodvessels, and at length obliterates them to such an extent as to atrophy and destroy the granulations themselves. The mucous membrane is now found to be paler than is natural, and to be hard and gristly, like the tissue of a cicatrix. The contractile tendency here is doubtless the same which we see in granulation of the eyelids, where it produces similar results; and, in addition, by diminishing the area of the conjunctive, draws in the edges of the lids until the ciliæ rub on the eye. Hence why entropium so often follows granular conjunctivitis. A perfectly analogous phenomenon is observed in granular urethritis. In every case, according to Desormeaux, there is present some contraction of the calibre of the urethra, which, even when slight, may be detected by a practised hand in introducing a sound, and in more aggravated cases constitutes stricture. Hence, the opinion that this trouble is usually due either to the acuteness of the first inflammation, or to the injudicious use of nitrate of silver by the surgeon, must be considered erroneous. Granular urethretis tends towards stricture in all cases, though the narrowing is not always great enough to trouble the patient in urination. The same contraction, when it is considerable in amount, shortens the urethra so much as to be an obstacle to erection, constituting the chronic form of chordee.

The granular disease, situated in the cervix uteri, is probably the cause of the well-known barrenness of strumpets, and of women whose husbands have been of loose habits. In the latter case, the woman produces perhaps one or two children, but by this time gets from her husband a granular metritis, which obstructs the os uteri, and effectually prevents further conceptions. It is in this way that licentiousness operates to restrict the growth of population, and correspondingly diminishes the progress and power of nations.

In conclusion, I would state, that I think the endoscope is a valuable addition to our resources in urethral diseases; but that examinations, by means of it, into the interior of the bladder, the cavities of tumors, and the tracks of fistulas, will be found rather matters of curiosity than of usefulness. I think, also, that in consequence of the dimming of the reflectors, by time and other deteriorations, it will only be satisfactory in ordinary hands when used with the addition of the magnesian light.

## Figures and Tables

**Fig.1 Fig.2. Fig 3 Fig.4 f1:**